# Designing patient-facing health information technologies for the outpatient settings: a literature review

**DOI:** 10.14236/jhi.v23i1.185

**Published:** 2016-04-06

**Authors:** Yushi Yang, Onur Asan

**Affiliations:** Department of Industrial Engineering, Clemson University, Clemson, SC, USA; Center for Patient Care and Outcomes Research, Division of General Internal Medicine, Department of Medicine, Medical College of Wisconsin, Milwaukee, WI, USA; Center for Patient Care and Outcomes Research, Division of General Internal Medicine, Department of Medicine, Medical College of Wisconsin, Milwaukee, WI, USA

**Keywords:** electronic health record (EHR), macroergonomics, patent-facing health information technology (HIT), screen sharing

## Abstract

**Introduction:**

The implementation of health information technologies (HITs) has changed the dynamics of doctor–patient communication in outpatient settings. Designing patient-facing HITs provides patients with easy access to healthcare information during the visit and has the potential to enhance the patient-centred care.

**Objectives:**

The objectives of this study are to systematically review how the designs of patient-facing HITs have been suggested and evaluated, and how they may potentially affect the doctor–patient communication and patient-centred care.

**Method:**

We conducted an online database search to identify articles published before December 2014 relevant to the objectives of this study. A total of nine papers have been identified and reviewed in this study.

**Results:**

Designing patient-facing HITs is at an early stage. The current literature has been exploring the impact of HITs on doctor–patient communication dynamics. Based on the findings of these studies, there is an emergent need to design more patient-centred HITs. There are also some papers that focus on the usability evaluation of some preliminary prototypes of the patient-facing HITs. The design styles of patient-facing HITs included sharing the health information with the patients on: (1) a separate patient display, (2) a projector, (3) a portable tablet, (4) a touch-based screen and (5) a shared computer display that can be viewed by both doctors and patients. Each of them had the strengths and limitations to facilitate the patient-centred care, and it is worthwhile to make a comparison of them in order to identify future research directions.

**Conclusion:**

The designs of patient-facing HITs in outpatient settings are promising in facilitating the doctor-patient communication and patient engagement. However, their effectiveness and usefulness need to be further evaluated and improved from a systems perspective.

## INTRODUCTION

Doctor-patient communication has been reported to have a profound effect on the outcome of care.^1,2^ The primary goals of doctor-patient communication are to facilitate interpersonal relationships, information exchange, and treatment plan decision-making.^3^ The patient health outcomes are significantly dependent on the effectiveness of doctor–patient communication.^4**–**6^ Patient participation depends on doctors, patients, and a number of contextual factors, which all contribute to the quality of care.^7**–**12^ Therefore, there has been an increased attention in the research regarding patient-centredness, engagement, involvement and empowerment.^5,6,13^ While achieving patient-centred care is challenging, numerous technologies have been developed to facilitate a trustful and collaborative experience for doctors and patients in the outpatient settings, such as health information technologies (HITs).

The use of computer and HITs, such as electronic health record (EHR), has changed the dynamics of doctor–patient communication.^6,14,15^ EHRs contain various kinds of data entry and review of patient health information as well as the record of communication between healthcare providers or even hospitals.^16^ Studies have reported positive impacts of HITs on patient care, such as the improvements in quality and efficiency of medical care, patient safety, biomedical information exchange and clinical decision making.^17**–**19^ However, the communication between doctors and patient is no longer a simple face-to-face communication. The research has shown that doctors may spent excessive time with HIT and may reduce doctor’s interaction time and eye contacts with the patients during the visit.^20,21^ Gazing at the computer screen excessively may lose the engagement and rapport with patients,^22,23^ because it would be difficult for doctors to divide their attention between the patient and the computer.^24^ To address these issues, some recent studies have explored strategies for the effective use of HIT to increase the patient engagement.^15,25,26^

Designing patient-facing HITs is one of the promising strategies. Some of the potential features of patient facing HITs are being more interactive^27^ and more efficient screen sharing with the patients.^28^ The research has shown that sharing numbers and visualized clinical information with the patients may increase the transparency of healthcare information and facilitates patients’ understanding of their health condition.^29,30^ Screen sharing might also facilitate patient-centred collaboration and patient activation.^6,15^ Patients have expressed a strong patient-centred attitude toward information sharing via EHRs during the communication.^31^

A review of the studies related to the patient-facing HITs design has not been done before, so there is a need to understand the current stage of related research activities, their values, effectiveness and barriers to patient-centred care. The objectives of this study are to systematically review papers, to investigate how patient-facing HITs have been suggested or evaluated and how they may potentially affect the outcomes of doctor–patient communication in outpatient settings. Based on a comparison of the benefits and limitations of different design styles, we aim to suggest future research directions. In this study, we particularly take a socio-technical perspective,^32,33^ and thus, the scope to understand the problem becomes holistic and systematic.

## METHODS

### Search strategy

The authors conducted an online database search to identify articles published before December 2014 relevant to the objectives of this study. The articles were included as indexed in three reference databases: Web of Science, PubMed and PsycINFO. Broad keyword searches were used to identify relevant articles in each database. Each search included three parts: (1) doctor–patient communication (e.g. ‘physician–patient discussion’, ‘doctor–patient communication’, ‘patient-centredness’, ‘communication’ and ‘patient–doctor collaboration’; (2) Patient-facing HIT (e.g. ‘information sharing’, ‘HIT information sharing’, ‘interactive computing’, ‘interactive solutions’, ‘human–computer interactions’, ‘technology for information sharing’ and ‘EHR sharing’); and (3) outpatient setting (e.g. ‘outpatient’, ‘primary care’, ‘exam room’, ‘emergency department’ and ‘specialty clinics’). We screened the search results by reviewing titles and abstracts after the initial search and removing duplicates. We identified additional papers by examining the included papers’ reference lists.

### Inclusion and exclusion criteria

The scope of this study was determined by inclusion and exclusion criteria. We included papers with a suggested or evaluated design of the patient-facing HIT in the outpatient settings. We excluded the following papers: (1) HIT and their impacts on the communication (this topic has been reviewed in other studies);^20,34**–**36^ (2) designs of patient-facing HIT applied to inpatient settings; (3) early papers published five or more years ago (prior to 2009) (designing patient-facing HIT is a just recent research topic with rapid changes, and therefore, early papers on this topic would lack enough time-lessness); (4) papers not in English; and (5) papers with a design for long-distance communication, such as the email systems, the telecommunication technology and online clinical consultation systems.

### Data analysis

We extracted key data from the selected papers that met the inclusion criteria based on the method description approach.^37^ This set included the title, author, purpose and key findings.^37^ After that, we did an inductive coding until recurrent themes emerged. This was an analytical process that allows the articles to be categorized based on factors that are arranged to compare and relevant to the research questions.^38^ Through the coding process, the following topics were explored as the important themes: paper objectives, study design, the doctor–HIT–patient communication dynamics and patient-facing HIT designs.

## RESULTS

### Literature search overview

**Figure 1:**
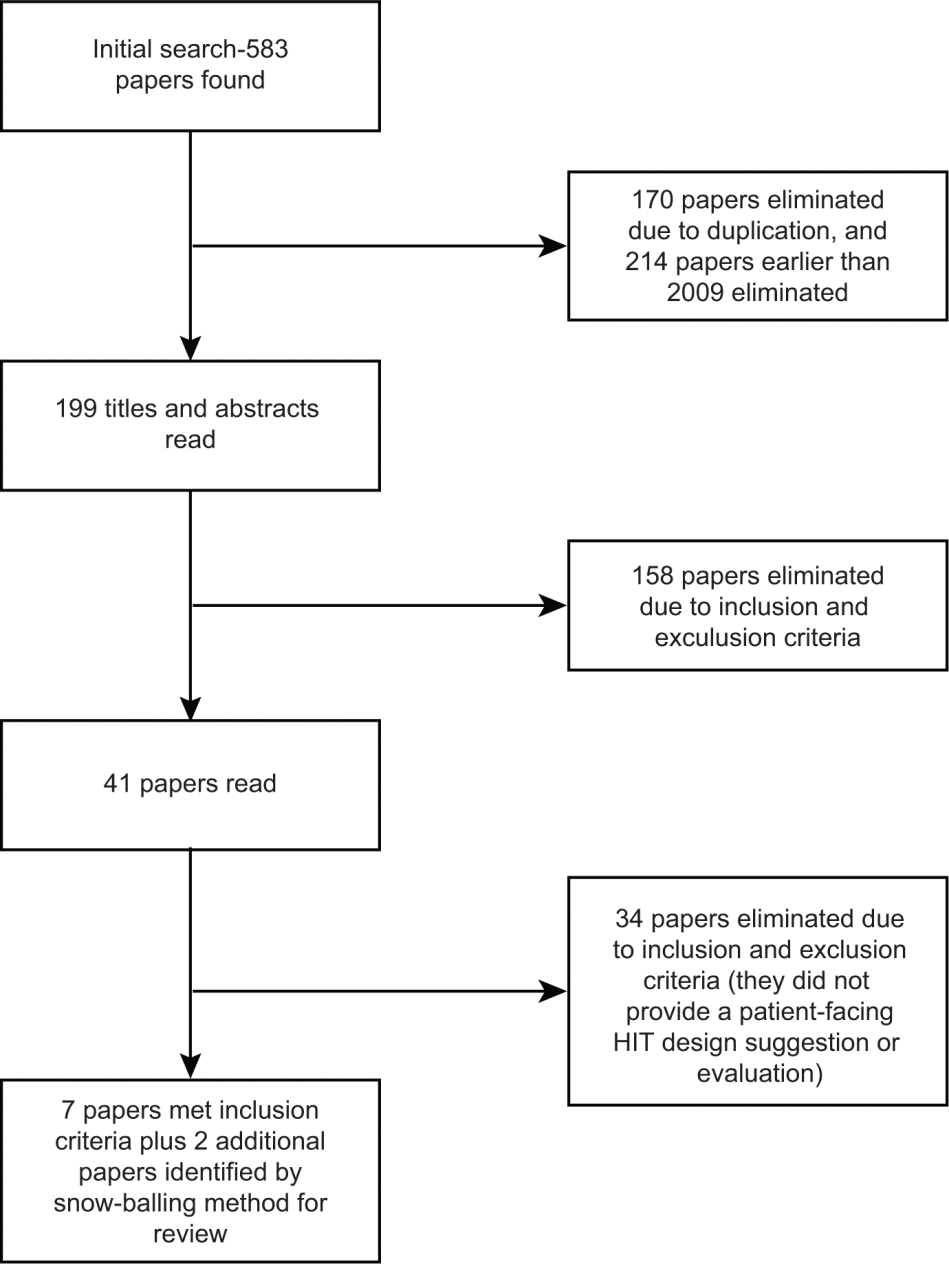
Flow diagram of the paper selection process

A total of 583 papers were found through the database search based on our search strategy. One hundred and seventy papers were removed due to duplication. After removing early papers published before 2009, 199 papers remained. We screened the remaining papers by comparing the titles and abstracts with the inclusion and exclusion criteria, leaving 41 papers that were fully qualified. After reading the entire paper, eight papers, which contained at least a design recommendation or evaluation of the patient-facing HITs, were included in the final results. Other papers were excluded based on the inclusion and exclusion criteria as described in the Method section. A reverse snow-balling method (reviewing the identified papers’ references) resulted in two additional papers. This resulted in a total of nine papers in this review (Figure 1). An overview of the papers can be found in Table 1.

### Paper objectives

Four papers were mainly contextual inquiries. Their aims were to understand the changes in dynamics of doctor–patient communication^39**–**42^ when EHRs were implemented in the outpatient settings. For example, some papers investigated how doctors communicate with patients while interacting with HIT.^39,40^ Others identified the challenges during communication when EHR is present in the room.^41,42^ They provided the basis for proposing the designs of patient-facing HITs. One paper was mainly a design description.^43^ It described a design concept of patient-facing HIT to enhance the doctor–patient communication.^43^ The four papers were mainly design evaluations.^44**–**47^ They presented the results of usability evaluations of the low-fidelity prototypes of patient-facing HITs.^44**–**47^

### Study design

The contextual inquiry papers used real-world observation methods, either video-recordings^39,41^ or shadowing.^40,42^ They also used the method of semi-structured interviews with patients only^40,42^ or all of the stakeholders involved in the design of healthcare work spaces, including clinicians, patients, architects and facility managers.^41^ The design description paper had no formal study design, though an informal contextual inquiry was conducted with an oncologist.^43^ The design evaluation papers tested the low-fidelity prototypes in simulated consultation settings,^44,45,47^ or the real clinical setting.^46^ Some collected quantitative data only using surveys and questionnaire^44,45^; others collected both quantitative and qualitative data using interviews, behavioral observations and questionnaires.^46,47^ The participants were the general public,^44,45,47^ or the patients and care providers.^46^

### The doctor–HIT–patient communication dynamics

Some major challenges regarding doctor–HIT–patient communication dynamics were reported in some of these studies. First, doctors spent more time with the computers and talked less with the patients during the medical consultation.^44^ Second, doctors had less eye contacts with the patients, making them feel ignored and less engaged.^39,40^ Third, computers created more opportunities for multitasking, fragmented attention and workflow disruptions during the communication.^39,42^ Fourth, sharing sensitive information with the patients via EHR screen created privacy issues and concerns, especially from the doctor’s perspective.^39,41^

While the impact of EHRs on doctor–patient communication might be related to doctors’ EHR use style, communication style, and workload, there might be other factors related to the sociotechnical aspect of the health care system. For example, in the current primary care exam room setting, there is triad interaction: active user of EHR (doctor), passive user of HIT (the patient) and the computer (HIT) itself, which mediates the doctor–patient communication and be used as a tool by the doctor in the visit.^39^ Therefore, when patients act most likely a passive user, with little opportunities to actively engage into receiving information from the EHR, the quality of conversation depend on more providers’ communication style and behavior of information sharing using the EHR screen.^39^ Another study reported that the frequent note taking and record checking on the computer displays with computers-on-wheel created some tension among the patients.^40^ Some younger patients also expressed their desire to see more technology-aided communication with their doctors.^41^ Furthermore, studies also reported that HITs have not been utilized with its full potential to facilitate doctor–patient communication in the visit due to various reasons, such as the lack of training and technical difficulties.^41^ The constraints of the physical positions, space and layouts of the clinical environment were also reported as potential barriers to use EHR as an efficient communication tool between doctors and patients in the visit.^42^

### Patient-facing HIT designs

In the reviewed papers, designs for patient-facing HIT were suggested and evaluated. Patient-facing HIT provided patients with a secondary view of EHR information. They were suggested or designed to share information with patients using different styles, such as a separate patient display,^39,46^ a projector,^42,44^ a portable tablet,^41,43,45^ a touch-based screen,^41,42,47,48^ or a shared computer display that can be viewed by both doctors and patients.^40^

The papers envisioned some potential benefits of suggested designs. For example, with a separate patient display, doctors can share clear and understandable patient-specific information and facilitate active engagement during the visit.^39,46^ With a projector to display images on surfaces, the space of the clinical workspace can be utilized to a large extent to facilitate a shared understandings during the doctor–patient communication.^42^ Projecting images on body and model may improve patient understanding of the condition.^44^ With a portable tablet and a touch-based screen on the wall, information can be shared in a way to support the communication.^41^ For example, showing a list of topic on the tablet interface can facilitate a proactive discussion and improve patient involvement.^43^ Showing videos or three-dimensional image instructions on a tablet can improve the patients’ understanding of clinical information.^45^ Showing charts and diagram on a large touch-based display can facilitate the collaboration between the doctors and the older patients.^47^ With a shared screen that can be rotated and reoriented to different angles, doctors may be able to show medical information to the patients while maintaining the level of information transparency.^40^

In the papers reviewed in this study, designs of patient-facing HITs have been proposed based on contextual inquiries and evaluated based on user studies. Some papers focused on the understanding of doctor–HIT–patient communication dynamics. They described the characteristics of technology use patterns during the doctor–patient communication. They also provided the basis to optimize the interactions of doctor–HIT–patient using patient-facing HIT designs. Some other papers focused on the evaluation of preliminary design prototypes of patient-centred HITs. They provided the evidence that sharing EHR information with patients enabled a mutual view of important information and improved the doctor–patient communication and patient engagement.

The proposed or evaluated designs of patient-facing HITs include: (1) a separate patient display, (2) a projector, (3) a portable tablet, (4) a touch-based screen and (5) a shared computer display, which can be used to view information by doctors and patients. While the current literature has envisioned the potential of patient-facing HITs on patient-centred care, a comparison of five suggested or evaluated design style have not been specified. To fill this gap, we did a comparison of both strengths and limitations of the five design styles of patient-facing HITs. The comparison was shown in Table 2.

Shared computer display or touch-based interface might provide the opportunity for both providers and patient to interact with the technology to access patient information together.^40,47^ A particular benefit of a touch-based interface is its large size, and therefore, information display, such as fonts and images, would be easier to interact and more clarity.^47^ However, Chen^40^ argued that the information transparency of a shared display or a touch-based interface might be inappropriate issues during certain phases of the outpatient medical consultation, because doctors may prefer not to share their private notes with patients through a shared computer screen.^39,40^ A separate patient display and a projector are the alternative design styles of patient-facing HIT, through which doctors can decide which information in the EHR to be shared with the patients.^39^ While they addressed the doctor’s privacy issues to an extent, they also introduced new barriers into the system, such as the costs and availability to implement the technology, the increased workload to interact with the technology and additional training needs for doctors to operate the technology. Moreover, showing patient-specific information on a tablet might increase the patient’s understanding of medical information^45^; however, with two separate interfaces (doctor’s computer and patient’s tablet) during the communication, doctors and patient may not have frequent eye contacts, which are essential to reach a mutual understanding and establish trusts.^49^ It might also be difficult for them to be on the same page during a communication when interacting with different interfaces with different contents. On the other hand, separate computer screens in the room might have the opportunity to have more patient-centred display. In this case, the doctor can pull up whatever data he want to share to the second screen, and they can both look at that screen and discuss the data. In this case, they will eliminate the clutter and nonuser friendly display of the main screen and prevent potential risk of privacy concerns.

Based on this literature review, we also identified several research opportunities that should be taken into account in the design of patient-facing HITs. The healthcare system is a complex sociotechnical system.^50^ That said, a good design must reconcile needs and preferences from multiple stakeholders involved in the system. Therefore, patient-facing HIT design must be proposed and evaluated from a systems perspective with the inquiries from both doctors and patients and even family members. Research in other areas has shown patients and doctors have different perceptions of the role of personal health records in the preventive health care.^51^ However, only one paper conducted a contextual inquiry from both doctor’s and patient’s sides.^41^ Also, only one paper evaluated the interactive design prototype with the two user groups.^46^ The lack of understanding from both sides makes it a challenge to holistically understand the problem from a system perspective and to propose a solution that is compatible with overall system goals.^52^ Besides that, based on the Systems Engineering Initiative for Patient Safety model,^32^ research has shown multiple system factors associated with different work system elements to influence doctors’ decision to share or not to share the screen.^53^ For example, a major obstacle for an active screen sharing might be the room layout or time restrictions in the visits.^54^ Also, showing the data on doctor’s EHR screen itself may not be helpful for the patient centredness because of its current design.^34^ They must be complemented by an interface that is designed specific to patients^55^ and accompanied by necessary explanations of what they see from the doctors. Therefore, to achieve a best patient-centred outcome with implementation a new tool, we must reconcile the needs and effects of all the elements in the entire system, such as the patients (their age, ability, disease, expectations, etc.), doctors (their specialty, preferences, concerns, sensitivity to privacies, etc.), the system settings, the physical environment and the organization and management (privacy, trainings, regulations, etc.).^32^ Besides, the design process must be integrated at different layers.^56^ For example, at a cognitive level, a design should not add mental workloads to the doctors and patients during the communication; at an individual level, a design must meet and satisfy the needs of both doctors and patients; and at an organizational level, a design must comply with the culture and norm of the work system.

### Call for future research

Future research is needed to compare the effects of the separate patient display, projectors, the portable tablet, the touch-based shared display and shared screen that can be viewed by doctors and patients on doctor-patient communication and patient outcomes in the long run in the outpatient settings. Particularly, the research of doctor–HIT–patient dynamics and the design of patient-faced HIT should be conducted from a systems perspective to meet the demands and satisfy the needs of both doctors and patients. The sociotechnical effects of the implemented design of patient-facing communication technologies need to be considered at multiple levels.

## CONCLUSION

In this review, we systematically reviewed the papers of the designs of patient-facing HITs and their effects on doctor–patient communication. Contextual inquires have been conducted to identify the needs for the design and user-centred research has been conducted to evaluate the proposed design. Based on the papers, designing patient-facing HIT in different styles might facilitate the doctor–patient communication in different ways. However, their effects, especially the sociotechnical effects, have not been holistically investigated from a systems perspective. Therefore, in the future, human factors researchers need to deeply understand the doctor–HIT–patient dynamics from both the doctor’s and patient’s perspectives. It is especially essential to investigate the sociotechnical systems outcome at different levels for the best patient-centred outcome.

## Figures and Tables

**Table 1:** Paper summaries

Paper Author	Title	Purpose	Key Findings
Asan and Montague, 2013	Technology-Mediated Information Sharing Between Patients and Clinicians in Primary Care Encounters	To understand technology-mediated information sharing between patients and clinicians in primary-care encounters.	There are three technology-mediated information-sharing styles: active information sharing, passive information sharing and technology withdrawal.
Chen et al, 2011	Unpacking Exam-room Computing: Negotiating Computer-use in Patient–physician Interactions	To examine the use of computer-on wheels and explore computer-based micro-negotiation in the exam rooms	There are three modes of micro-negotiation: exclusive viewing, collaborative viewing and neutral viewing, which achieve different goals.
Fonville et al, 2010	Exploring the Use of Technology in Healthcare Spaces and Its Impact on Empathic Communication	To investigate how the design of healthcare spaces and the technologies inside affect doctor–patient interaction and communication, in order to inform new design.	Doctor-patient communications face the challenges of limited time and resources, inefficient information sharing and the lack of empathic communication.
Gonzales and Riek, 2012	A Shared Interface to Improve Oncologist–Patient Communication	To propose a solution utilizing a shared mobile device to facilitate patient-physician communication during cancer discussions.	This pervasive technology promotes patient–physician discussion and understanding between both parties.
Ni et al, 2011	AnatOnMe: Facilitating Doctor–Patient Communication Using a Projection-Based Handheld Device	To present the design, development and evaluation of AnatOnMe, a projection-based handheld device designed to facilitate medical information exchange	AnatOnMe projects medical images on any surface. Empirical evidence suggested it can support information exchange and facilitate the doctor–patient communication
Piper and Hollan, 2013	Supporting Medical Communication for Older Patients with a Shared Touch-Screen Computer	To explore how a large horizontal touch-screen (i.e. a surface computer) may suit the needs of older patients and facilitate the doctor–patient interview process.	Participants suggested that having a shared view of one’s medical records, especially charts and images, would enhance communication with their doctor and aid understanding.
Schooley et al, 2015	Patient-Provider Communications in Outpatient Clinic Settings: A Clinic-Based Evaluation of Mobile Device and Multimedia Mediated Communications for Patient Education	To understand how information-assisted video and 3D image instructions influence the patients’ understanding of information about their condition and their attitudes towards their healthcare providers.	Patients found the computer-assisted instructional systems for patients helpful to understand their conditions, and found that the system made it easier to communicate with their healthcare providers.
Unruh et al, 2010	Transforming Clinic Environments into Information Workspaces for Patients	To understand how patients interact with information and manage information work in clinic environments and to propose design directions based on the findings.	Patients emphasized the importance of interaction time with their clinicians during clinic visits. They also have fragmented attention and heightened stress in clinic environments.
Wilcox et al, 2010	Designing Patient-Centric Information Displays for Hospitals	To explore how a patient-centred information display can deliver useful information to a patient during the course of an Emergency Department visit.	The subjective responses to in-room displays were overwhelmingly positive, and guidelines regarding specific information types, privacy, use cases, and information presentation techniques were elicited.

**Table 2:** 

Design Description	Strengths	Limitations
**A separate patient display**	Doctors have the power to control over what types of contents in the EHR may be shared with patients	Technology availability, reliability and cost; may increase doctor’s workload; and additional training required.
**A projector**	Doctors have the power to control over what types of contents in the EHR may be shared with patients and easy to move.	Technology availability, reliability and cost; May increase doctor’s workload; Additional training required.
**A portable tablet**	Easy to move, patients have more control when interacting with the tablet, and can access more individualized information.	Doctors and patients may not be on the same page during communication.
**A touch-based screen**	Doctors and patients can easily interact with the screen together and the data is clearly shown with large font size and visualization.	Information transparency without reservation might not be appropriate at certain situations and some patients may feel the large screen intimidating.
**A display a shared computer display that can be viewed by both doctors and patients**	Patients can be more engaged during the consultation, doctors and patients can easily be on the same page, and information transparency may be maintained and reserved by the doctors.	Information might not be easily viewed with clarity by both doctors and patients and the layout of the physical space may be the barrier for viewing on the same screen.
